# Soluble CD40L is associated with increased oxidative burst and neutrophil extracellular trap release in Behçet’s disease

**DOI:** 10.1186/s13075-017-1443-5

**Published:** 2017-10-19

**Authors:** Sandro Félix Perazzio, Paulo Vitor Soeiro-Pereira, Viviane Cardoso dos Santos, Marlon Vilela de Brito, Bruno Salu, Maria Luiza Vilela Oliva, Anne Margherite Stevens, Alexandre Wagner Silva de Souza, Hans D. Ochs, Troy R. Torgerson, Antonio Condino-Neto, Luis Eduardo Coelho Andrade

**Affiliations:** 1 Division of Rheumatology, Escola Paulista de Medicina, Federal University of Sao Paulo, Rua Botucatu 740, 3° Andar, 04023-062 Sao Paulo, SP Brazil; 2 Fleury Group – Research and Development, Avenida General Valdomiro de Lima, 508, 04344-070 Sao Paulo, SP Brazil; 3 Seattle Children’s Research Institute, University of Washington and Center for Immunity and Immunotherapies, 1900 9th Avenue, JMB-7, Seattle, WA 98101 USA; 4 Department of Immunology, Institute of Biomedical Sciences, University of Sao Paulo, Avenida Professor Lineu Prestes, 2415, 03178-200 Sao Paulo, SP Brazil; 5 Department of Pathology, Federal University of Maranhao, Avenida dos Portugueses, 65065-545 Sao Luiz, MA Brazil; 6 Department of Biochemistry and Molecular Biology, Escola Paulista de Medicina, Federal University of Sao Paulo, Rua Três de Maio, 100, 5° Andar, 04044-020 Sao Paulo, SP Brazil

**Keywords:** Behçet’s disease, Neutrophil activation, sCD40L, CD40L pathway, Oxidative burst, Neutrophil extracellular traps

## Abstract

**Background:**

Studies have suggested that soluble factors in plasma from patients with active (aBD) and inactive (iBD) Behçet’s disease (BD) stimulate neutrophil function. Soluble CD40 ligand (sCD40L) is an important mediator of inflammation in BD. Its expression and effect on neutrophil oxidative burst and neutrophil extracellular trap (NET) release have not been characterized. In this study, we sought to investigate the role of plasma and the CD40L pathway on NET release and the oxidative burst profile in patients with aBD and iBD.

**Methods:**

Neutrophils and peripheral blood mononuclear cells (PBMCs) were obtained from patients with aBD (*n* = 30), patients with iBD (*n* = 31), and healthy control subjects (HCs; *n* = 30). sCD40L plasma concentration was determined in individual samples. A pool of plasma for each group was created. In some experiments, plasma pools were treated with recombinant CD40 (rhCD40-muIg) for sCD40L blockade. NET release and H_2_O_2_/O_2_^−^ production were determined after stimulation with phorbol 12-myristate 13-acetate, sCD40L, or plasma pool. Flow cytometric analysis was performed to evaluate the expression of (1) CD40, Mac-1, and phosphorylated NF-κB p65 on neutrophils and monocytes and (2) CD40L on activated T cells and platelets. CD40L gene expression in PBMCs was determined by qRT-PCR.

**Results:**

sCD40L plasma levels were significantly higher in patients with iBD (median 17,234, range 2346–19,279 pg/ml) and patients with aBD (median 18,289, range 413–19,883 pg/ml) than in HCs (median 47.5, range 33.7–26.7 pg/ml; *p* < 0.001). NET release was constitutively increased in BD compared with HC. NET release and H_2_O_2_/O_2_^−^ were higher after stimulation with sCD40L or BD plasma and decreased after sCD40L blockade. Mac-1 expression was constitutively increased in neutrophils of patients with aBD (88.7 ± 13.2% of cells) and patients with iBD (89.2 ± 20.1% of cells) compared with HC (27.1 ± 18.8% of cells; *p* < 0.01). CD40 expression on phagocytes and CD40L expression on platelets were similar in the three groups. PBMCs as well as nonactivated and activated CD4^+^ T cells from patients with BD showed higher CD40L expression.

**Conclusions:**

Plasma from patients with aBD exerts a stimulus on NET release and oxidative burst, probably induced by sCD40L.

**Electronic supplementary material:**

The online version of this article (doi:10.1186/s13075-017-1443-5) contains supplementary material, which is available to authorized users.

## Background

The pathogenesis of Behçet’s disease (BD) is unknown; however, there are pieces of evidence pointing to increased neutrophil activation in patients with BD that can be clinically correlated with the pathergy phenomenon [1]. Phagocyte functional abnormalities possibly involved in BD include cytokine production [2], oxidative burst [3], and phagocytic [4, 5] and microbicidal activity [6], but divergent results have been reported by different authors [1, 4, 5, 7, 8]. Our group recently reported on the heterogeneity in BD pathophysiology by showing that only patients with severe active BD exhibit evidence of constitutive neutrophil activation judged on the basis of oxidative burst activity and cytokine production [9].

Although several pieces of evidence point to participation of neutrophils in BD, evaluation of neutrophil extracellular trap (NET) formation in BD has not been investigated. Moreover, it is unclear whether phagocyte activation in patients with BD occurs constitutively, mainly in those carrying HLA-B51 [3], or if it is secondary to an as yet unknown stimulus [10, 11]. Also, has not been defined whether the neutrophil hyperactive state is induced by some soluble serum or tissue factor or if BD neutrophils or other cell subtypes produce soluble inflammatory mediators carried by plasma, as previously suggested for specific phagocyte functions, such as chemotaxis [10] and oxidative burst [11].

Soluble CD40 ligand (sCD40L) is a cleaved form of CD40L present in the plasma. Its membrane-bound analogue is involved in immunoglobulin isotype switching, B-cell differentiation, antigen-presenting cell activation, T-cell modulation, and thrombocyte aggregation [12]. The major source of sCD40L is activated platelets, but activated T cells, macrophages, endothelial cells, and smooth muscle cells also contribute to sCD40L synthesis and shedding [13].

Fernández-Bello et al. [14] showed higher levels of plasma and platelet-derived sCD40L in a group of patients with BD, although no differences in platelet activation or platelet-leukocyte aggregate formation were observed. In normal mouse neutrophils, sCD40L strongly stimulates the oxidative burst via a CD40-dependent phosphoinositide 3-kinase/NF-κB pathway [15]. Additionally, platelet-derived sCD40L stimulates expression of the leukocyte-specific β_2_-integrin Mac-1 in neutrophils and thereby further promotes neutrophil adhesion and migration [15]. Therefore, it is possible that elevated plasma sCD40L contributes to the characteristic phagocyte hyperactivity in BD.

It has been postulated that BD may be a form of autoinflammatory disease [16]. Interestingly, although some pieces of evidence point to neutrophil hyperactivation in BD, it is not clear if this is a primary (constitutive) phenomenon or if it is secondary to putative soluble factors. We hypothesize that sCD40L carried at increased levels by the plasma of patients with BD is associated with systemic phagocyte activation. In the present study, we investigated the role of plasma from patients with BD and sCD40L on NET release and oxidative burst profiles in neutrophils and peripheral blood mononuclear cells (PBMCs) from patients with active Behçet’s disease (aBD) and patients with inactive Behçet’s disease (iBD).

## Methods

### Study subjects and diagnostic criteria

This study included three main subject groups: (1) 30 healthy control subjects (HC), (2) 31 patients with iBD, and (3) 30 patients with aBD. All patients with BD met the International Study Group for Behçet’s Disease criteria [17]. Active BD was defined as a score ≥ 2 and iBD as a score of zero on the Simplified Behçet’s Disease Current Activity Form adapted for Portuguese by the Sociedade Brasileira de Reumatologia (sBR-BDCAF) [18]. The HC group comprised healthy adult volunteers from the hospital staff with no evidence or family history of autoimmune disease or immunodeficiency. Additionally, for some experiments, we used samples derived from 25 patients with sepsis (PSs), 27 patients with inactive pediatric systemic lupus erythematosus (iSLE), and 27 patients with active pediatric systemic lupus erythematosus (aSLE). The PS group, a positive control group for neutrophil activation, consisted of adult inpatients meeting the sepsis criteria of the American College of Chest Physicians/Society of Critical Care Medicine Consensus Conference [19]. The SLE group consisted of pediatric patients meeting the American College of Rheumatology criteria for SLE [20]. Patients scoring ≥ 6 on the Systemic Lupus Erythematosus Disease Activity Index [21] were considered to have active disease, and those scoring zero were considered to have inactive disease. The study was approved by the institutional ethics review boards of Universidade Federal de São Paulo (CEP 0013/11) and Seattle Children’s.

### Plasma pools

Plasma pools for each group were assembled by mixing plasma samples derived from heparinized blood drawn from each subject enrolled in the study. Additionally, plasma from four different X-linked hyper immunoglobulin M patients collected at the immunology outpatient clinic of the University of Sao Paulo was used to constitute a pool of hyper immunoglobulin M plasma (HIgM). All pools were stored in aliquots at −80 °C.

### RNA extraction and complementary DNA synthesis

RNA was isolated by standard RNA extraction using TRIzol® Reagent (Life Technologies, Carlsbad, CA, USA) and transformed into complementary DNA (cDNA) by using the SuperScript One-Step RT-PCR System with Platinum Taq DNA polymerase (Life Technologies) using a thermocycler (Eppendorf, Hamburg, Germany).

### Quantification of cytokine and soluble receptors in plasma

After donation, blood samples were immediately spun at 2500 rpm for 15 minutes at room temperature, and 2-ml plasma aliquots with 40 μl of dipeptidyl peptidase-4 protease inhibitor (Merck Millipore, Billerica, MA, USA) were stored at −80 °C. All plasma samples were thawed only once for determination of the concentration of 13 soluble receptors (HSCR-32 K; Merck Millipore) and 64 cytokines (HCYTOMAG-60 K and HCYP2MAG-62 K; Merck Millipore) by addressable laser bead immunoenzyme assay according to the manufacturer’s specifications and read using the Luminex MAGPIX® System 40-072 (Merck Millipore). sCD40L plasma levels were subsequently assessed by enzyme-linked immunosorbent assay (ELISA) using the Human sCD40L Platinum ELISA kit (eBioscience, San Diego, CA, USA) according to the manufacturer’s specifications and read at λ = 450/570 nm in a VICTOR X3 2030 multilabel ELISA reader (PerkinElmer, Singapore).

### Hydrogen peroxide and superoxide anion quantification

Neutrophils and PBMCs were separated by density gradient separation using dextran and Ficoll-Hypaque 1077, respectively. Cells were stimulated with (1) 30 ng/ml phorbol 12-myristate 13-acetate (PMA; Sigma-Aldrich, St. Louis, MO, USA), (2) 50 μl of pooled plasma (from HCs, patients with iBD, or patients with aBD) with or without 2 μg/ml recombinant CD40 (rhCD40-muIg; (R&D Systems, Minneapolis, MN, USA), (3) 50 μl of pooled plasma from HIgM patients, or (4) 100 ng/ml rh-sCD40L (Life Technologies). Hydrogen peroxide and superoxide production was determined by luminol/lucigenin chemiluminescence intensity and quantified for 2 h at 37 °C during the readout in the VICTOR X3 2030 multilabel plate reader as described previously [22, 23]. Each well was read for 0.5 seconds every 50 seconds, and the results were expressed as a kinetic curve of relative light units on the *x*-axis and time in minutes on the *y*-axis. The analysis was based on the AUC.

### Expression of nicotinamide adenine dinucleotide phosphate oxidase components

Gene expression of nicotinamide adenine dinucleotide phosphate (NADPH) oxidase components was determined by qRT-PCR as described previously [22]. (Primers are shown in Additional file 1: Table S1.) Data were acquired and analyzed using a ViiA 7 RT-PCR system (Thermo Fisher Scientific, Waltham, MA, USA).

Protein expression of NADPH oxidase subunit protein expression was determined by flow cytometry. Briefly, 100 μl of whole blood was diluted with 100 μl of PBS and incubated with 30 ng/ml PMA or 50 μl of pooled plasma (from HCs, patients with iBD, or patients with aBD) for 6 h in a 5% CO_2_ atmosphere at 37 °C. For gp91-phox and p22 subunit evaluation, the samples were stained with 2 μl of anti-gp91-phox-fluorescein isothiocyanate (Santa Cruz Biotechnology, Dallas, TX, USA) and 2 μl of anti-p22-phycoerythrin (PE) (Santa Cruz Biotechnology), respectively, for 30 minutes in the dark. For p40, p47, and p67 subunit evaluation, the samples were initially fixed with 50 μl of BD Cytofix fixation buffer (BD Biosciences, San Jose, CA, USA) and then permeabilized with 50 μl of BD Phosflow Perm Buffer III (BD Biosciences) according to the manufacturer’s instructions. Then, the samples were stained with 2 μl of anti-p40-AF488 (Santa Cruz Biotechnology), 2 μl of anti-p47-AF647 (Santa Cruz Biotechnology), and 2 μl of anti-p67-PE (Santa Cruz Biotechnology). Using a FACSAria III flow cytometer (BD Biosciences), we observed that monocytes and neutrophils were gated by forward and side scatter. Acquired data were analyzed using FlowJo software (FlowJo, Ashland, OR, USA).

### Protein expression of NF-κB phosphorylated p65 subunit

Assessment of NF-κB p65 subunit phosphorylation was performed by flow cytometry as previously described [24] following incubation with 30 ng/ml PMA or 50 μl of pooled plasma (from HCs, patients with iBD, or patients with aBD) for 15 minutes in a 5% CO_2_ atmosphere at 37 °C.

### NET formation

NET release was determined as previously described [25] after stimulation with 30 ng/ml PMA, 100 ng/ml rh-sCD40L, or 50 μl of pooled plasma (from HCs, patients with iBD, or patients with aBD) with or without 2 μg/ml rhCD40-muIg. Cells were stained with primary anti-histone H2A/B (kindly provided by Dr. Arturo Zychlinsky, Max Planck Institute, Berlin, Germany) and anti-human neutrophil elastase monoclonal antibodies (Abcam, Cambridge, UK). Bisbenzimide (Life Technologies) was used for DNA labeling. Three random microscopic fields (×400 magnification) were photographed with a × 40 or × 63 lens objective and 17, 20, and 21 high-efficiency filters for each fluorochrome, respectively. Total NET area in each photo was calculated using ZEN lite 2012 (black edition) software (Carl Zeiss, Oberkochen, Germany).

### CD40L protein and gene expression

CD40L protein expression was determined by flow cytometry as previously described [14, 26]. Platelets were obtained and assessed by flow cytometry for CD40L protein expression before and after stimulation with 0.2 U/ml thrombin for 5 minutes at 37 °C in a 5% CO_2_ atmosphere [14]. CD40L protein expression in monocytes and in CD4^+^ and CD8^+^ T lymphocytes was determined by flow cytometry before and after stimulation with 1.5 μM ionomycin (Sigma-Aldrich) and/or 25 ng/ml PMA [26]. CD40L gene expression in PBMCs was determined before and after stimulation with 10 ng/ml PMA for 3 h using the TaqMan® Gene Expression qRT-PCR Assay for CD40L (Thermo Fisher Scientific). cDNA levels were normalized to the expression of glyceraldehyde 3-phosphate dehydrogenase (Thermo Fisher Scientific).

### Statistical analysis

Continuous variables were evaluated using the Kolmogorov-Smirnov test and analyzed using Student’s *t* test (normal distribution) or the Mann-Whitney *U* test (nonparametric distribution). Qualitative parameters were analyzed using the chi-square test and Fisher’s exact test when appropriate. Multiparametric analyses were conducted with one-way analysis of variance (ANOVA) followed by Bonferroni posttests when appropriate. The statistical inference level was set at 0.05.

## Results

### Study subject characteristics

The female-to-male ratios were 14:16 in the aBD group and 15:16 in the iBD group, with ages ranging from 20 to 57 years (36.13 ± 11.02) and from 20 to 55 years (40.96 ± 9.72), respectively. The female-to-male ratios were 18:12 in the HC group, 14:11 in the PS group, 18:9 in the iSLE group, and 22:5 in the aSLE group, with age ranges varying from 24 to 62 years (35.63 ± 9.49), 20 to 82 years (46.00 ± 25.76), 6 to 18 years (14.32 ± 2.96), and 7 to 20 years (15.12 ± 3.48), respectively. The HIgM plasma pool was constituted of plasma from four X-linked hyper-IgM male patients aged 4, 5, 7, and 8 years. Patients with BD and control subjects did not differ regarding gender distribution. The HC, iBD, aBD, and PS groups did not differ regarding age distribution. The iSLE, aSLE, and HIgM groups were significantly younger than the other groups.

Miscellaneous comorbidities (osteoarthritis, osteopenia/osteoporosis, glaucoma, arterial hypertension, dyslipidemia, chronic obstructive pulmonary disease, and epilepsy, among others) were equally present in patients with iBD (*n* = 22; 70.96%) and patients with aBD (*n* = 19; 63.33%) (*p* = 0.59). The clinical characteristics and therapeutic regimens of patients with BD are shown in Table 1. There were no significant differences between patients with aBD and patients with iBD regarding previous disease manifestations. All patients were under treatment at the time of the study. As expected, the number of patients with aBD receiving cyclophosphamide or high doses of corticosteroids was higher than among patients with iBD.

**Table 1:** Distribution of patients with inactive and active Behçet’s disease, according to clinical manifestations and medication use

	iBD (*n* = 31) (%)		aBD (*n* = 30) (%)	
	Current	Previous	Current	Previous
Clinical manifestations				
Oral ulcers	0	31 (100)	21 (70.0)	30 (100)
Genital ulcers	0	28 (90.3)	5 (16.7)	24 (80.0)
Anterior uveitis	0	14 (45.2)	11 (36.7)	8 (26.7)
Posterior uveitis	0	10 (32.3)	7 (23.3)	11 (36.7)
Other ocular manifestations^a^	0	5 (16.1)	4 (13.3)	5 (16.7)
Pseudofolliculitis	0	18 (58.1)	6 (20.0)	11 (36.7)
Erythema nodosum	0	15 (48.4)	8 (26.7)	14 (46.7)
CNS	0	7 (22.6)	5 (16.7)	5 (16.7)
Gastrointestinal	0	2 (6.5)	0	1 (3.3)
Cardiovascular	0	5 (16.1)	3 (10.0)	4 (13.3)
Pathergy	5 (16.1)		2 (6.7)	
Others^b^	0	13 (41.9)	5 (16.7)	9 (30.0)
Disease duration, years, median/minimum–maximum	9/1 – 30		6/0 – 19	
Age at BD onset, years, mean (SD)	30.1 ± 9.6		28.7 ± 12.0	
sBR-BDCAF, median/minimum–maximum	0/0 – 0		3/2 – 4	
Medications	26 (83.9)	29 (93.5)	28 (93.3)	23 (76.7)
Colchicine	14 (45.2)	11 (35.5)	15 (50.0)	5 (16.7)
Azathioprine	14 (45.2)	10 (32.5)	16 (53.3)	4 (13.3)
Thalidomide	2 (6.5)	7 (22.6)	3 (10.0)	3 (10.0)
Methotrexate	3 (9.7)	6 (19.4)	1 (3.3)	3 (10.0)
Cyclophosphamide	0*	7 (22.6)	5 (16.7)^c^	2 (6.7)
Penicillin G benzathine	4 (12.9)	7 (22.6)	4 (13.3)	4 (13.3)
Anti-TNF	5 (16.1)	0	1 (3.3)	1 (3.3)
Corticosteroids				
High dose	5 (16.1)^d^	13 (41.9)	15 (50.0)^d^	12 (40.0)
Low dose	5 (16.1)	18 (58.1)	6 (20.0)	10 (33.3)
Cyclosporine	1 (3.2)	6 (19.4)	6 (20.0)	5 (16.7)
Pentoxifylline	2 (6.5)	2 (6.5)	1 (3.3)	2 (6.7)
Minocycline	0	3 (9.7)	0	0
Dapsone	0	0	0	1 (3.3)
Chlorambucil	0	2 (6.5)	0	1 (3.3)
Sulfasalazine	0	1 (3.2)	0	0

*Abbreviations: aBD* Active Behçet’s disease, *BD* Behçet’s disease, *CNS* Central nervous system, *iBD* Inactive Behçet’s disease, *sBR-BDCAF* Simplified Behçet’s Disease Current Activity Form adapted for Portuguese by the Sociedade Brasileira de Reumatologia, *TNF* Tumor necrosis factor

^a^Included cases with optic neuritis, vitreous hemorrhage, sudden blindness, and so forth

^b^Other manifestations: BD-induced dementia, arthralgia/arthritis, folliculitis decalvans, and so forth

^c^ *p* < 0.05

^d^ *p* < 0.01

### NET release is increased by neutrophils from patients with BD and is further stimulated by plasma from patients with BD

**Fig. 1:**
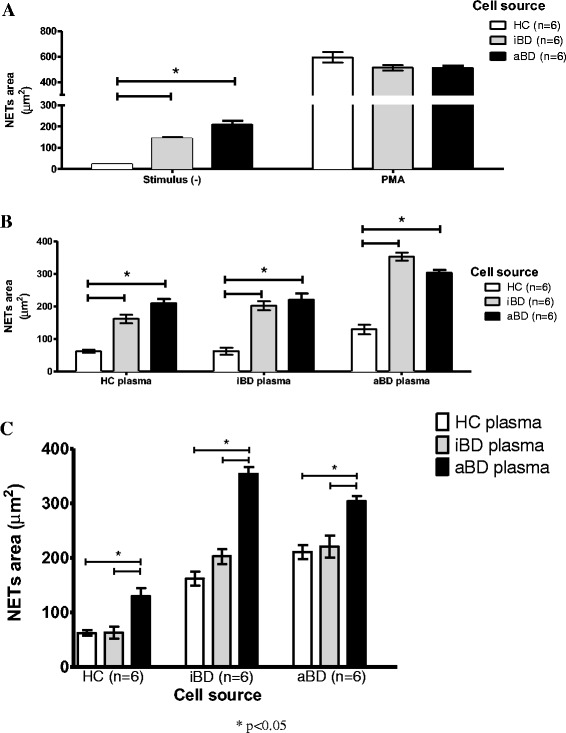
Neutrophil extracellular trap (NET) release is increased in Behçet’s disease (BD). **a** Resting neutrophils from healthy control subjects (HCs; *n* = 6), patients with active Behçet’s disease (aBD; *n* = 6), patients with inactive Behçet’s disease (iBD; *n* = 6), patients without stimulus, and after phorbol 12-myristate 13-acetate stimulation. **b** Effect of plasma from HCs, patients with iBD, and patients with aBD on NET release by neutrophils from HCs and patients with BD. (**c**) Plasma from patients with aBD exerted a stronger stimulus on NET formation by neutrophils from HC and patients with BD. Bars represent mean ± SE. Statistical analysis was performed with one-way analysis of variance and the Bonferroni posttest

**Fig. 2:**
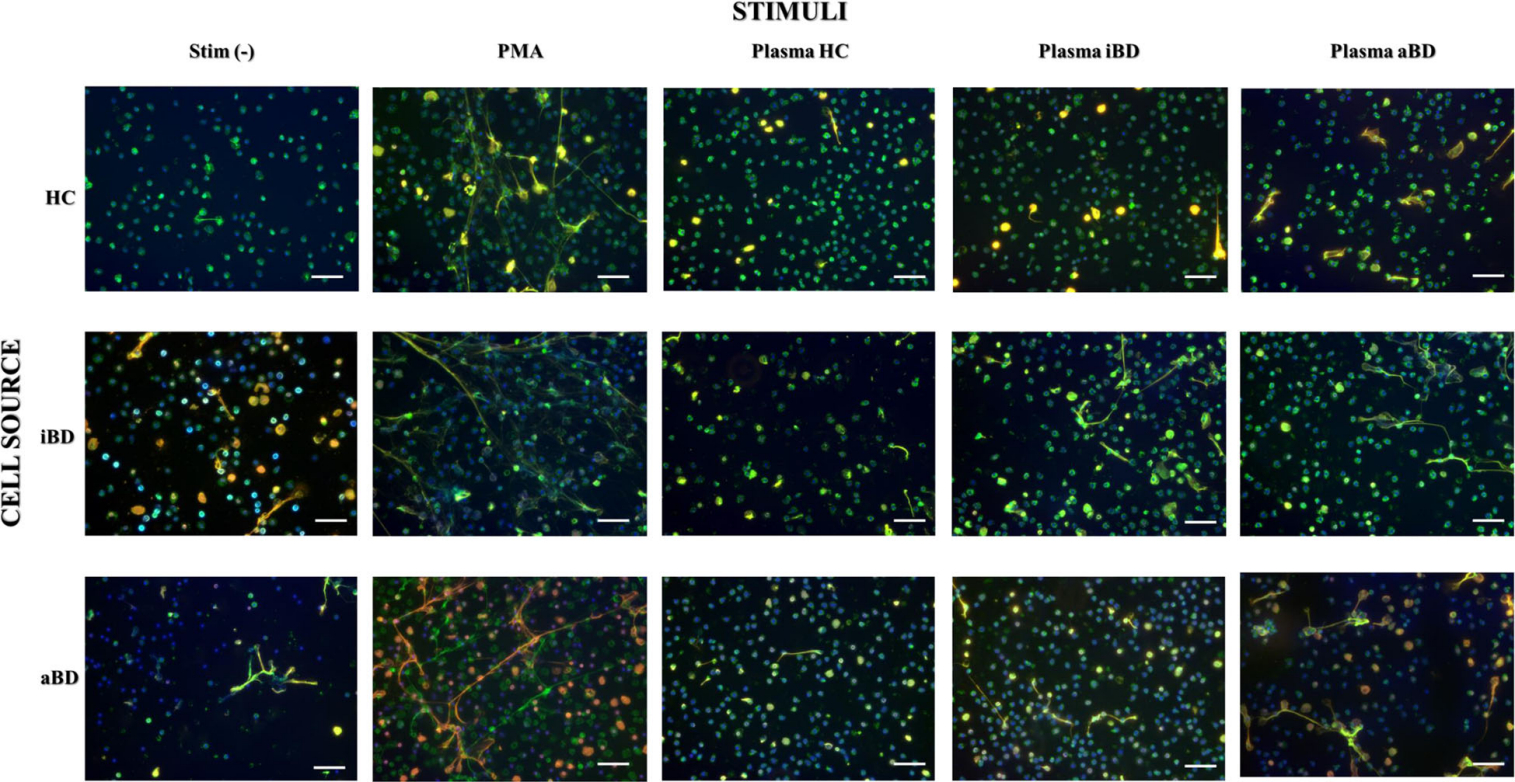
Neutrophil extracellular trap (NET) release stimulated by phorbol 12-myristate 13-acetate (PMA) or by a pool of plasma from healthy control subjects (HCs), patients with inactive Behçet’s disease (iBD), and patients with active Behçet’s disease (aBD). Immunofluorescence photomicroscopy illustrates the effect of plasma from patients with Behçet’s disease (BD) in normal and BD neutrophils. *White bars* indicate scale = 50 μm; *green* = histone H2A/B; *red* = human neutrophil elastase; *blue* = chromatin

Neutrophils from patients with BD showed a higher rate of spontaneous NET release than those from HCs, although no difference was observed after stimulation with PMA (Fig. 1a). When stimulated with plasma, neutrophils from patients with BD released more NET than HCs’ neutrophils (Fig. 1b). Moreover, NET release was more strongly stimulated by plasma from patients with aBD than by plasma from HCs or patients with iBD (Fig. 1c). Figure 2 shows photomicrographs illustrating the results.

### Plasma from patients with BD stimulates oxidative burst and NADPH oxidase protein expression

**Fig. 3:**
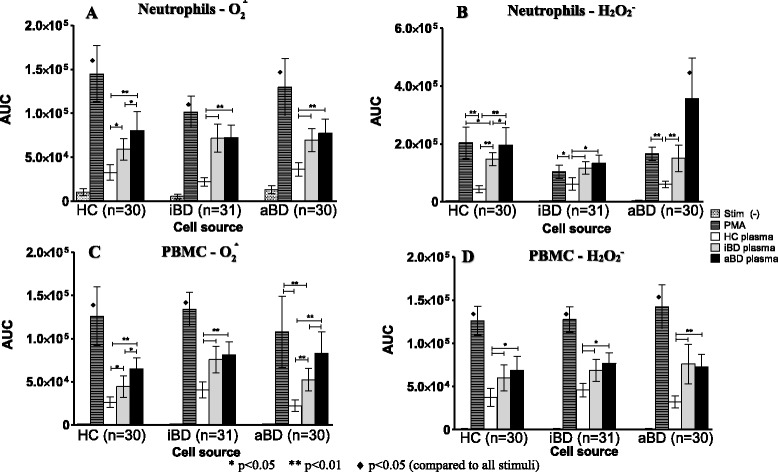
Plasma from patients with Behçet’s disease (BD) stimulates phagocyte oxidative burst activity. Superoxide (**a** and **c**) and hydrogen peroxide (**b** and **d**) production by neutrophils (**a** and **b**) and peripheral blood mononuclear cells (PBMCs) (**c** and **d**) from healthy control subjects (HCs; *n* = 30), patients with active Behçet’s disease (aBD; *n* = 30), and patients with inactive Behçet’s disease (iBD; *n* = 31) after human plasma or phorbol 12-myristate 13-acetate stimuli, calculated by the AUC. In general, BD plasma exerted a strong stimulus to oxidative burst by phagocytes from HCs, patients with iBD, and patients with aBD. Bars represent mean ± SE. Statistical analysis was performed by one-way analysis of variance with the Bonferroni posttest

Superoxide and hydrogen peroxide production in resting phagocytes was similar in patients with BD and HCs. Plasma from patients with BD, but not from HCs, stimulated oxidative burst in phagocytes from normal individuals and patients with BD (Fig. 3a–d). Interestingly, neutrophils from HCs produced more superoxide (Fig. 3a) and hydrogen peroxide (Fig. 3b) after stimulation with aBD plasma than with iBD and HC plasma. In general, PMA stimulated higher superoxide production in neutrophils than any other stimulus; however, hydrogen peroxide production by aBD neutrophils was higher after aBD plasma than after PMA stimuli, and hydrogen peroxide production by iBD neutrophils after PMA stimulation was equivalent to that observed with iBD or aBD plasma stimuli.

Similar results were also observed in PBMC assays, in which superoxide (Fig. 3c) and hydrogen peroxide production (Fig. 3d) were higher after BD plasma stimulation than after HC plasma stimulation. Remarkably, aBD plasma exerted a stronger effect than iBD plasma on superoxide production by PBMCs from HCs and were even comparable to PMA on aBD cells (Fig. 3c). In all the other comparisons, reactive oxygen species production by PMA-stimulated PBMCs was higher than with any other stimulus.

**Fig. 4:**
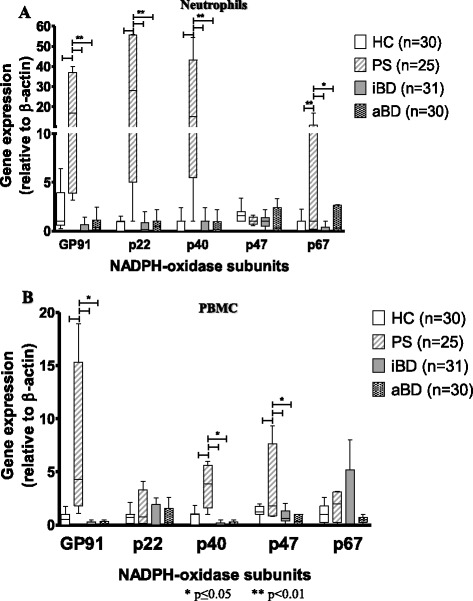
Gene expression of the five nicotinamide adenine dinucleotide phosphate (NADPH) oxidase subunits (gp-91-phox, p22, p40, p47, and p67) in resting neutrophils (**a**) and peripheral blood mononuclear cells (PBMCs) (**b**) from healthy control subjects (HCs; *n* = 30), patients with sepsis (PSs; *n* = 25), patients with inactive Behçet’s disease (iBD; *n* = 31), and patients with active Behçet’s disease patients (aBD; *n* = 30). Statistical analysis was performed using the Kruskal-Wallis test with the Mann-Whitney posttest

To determine whether the increased production of reactive oxygen species by BD neutrophils could be a result of increased expression of the NADPH oxidase subunits, we evaluated the level of subunit expression by both messenger RNA (mRNA) and protein analysis. Expression of each NADPH oxidase subunit gene was equivalent in resting neutrophils (Fig. 4a) and PBMCs (Fig. 4b) from HCs, patients with iBD, and patients with aBD. Cells from a group of 25 patients with severe sepsis were used as a positive control and, as expected, exhibited higher expression of gp91-phox, p22, p40, and p67 NADPH oxidase subunits. As for NADPH oxidase protein, the expression of the five subunits in neutrophils and monocytes increased significantly after stimulation with aBD or iBD plasma. Interestingly, plasma from HCs also caused an increase in NADPH oxidase protein expression, but plasma from patients with BD had a significantly stronger effect (Table 2).

**Table 2:** Nicotinamide adenine dinucleotide phosphate oxidase subunit protein expression in neutrophils and monocytes

Cell source	Stimulus				
	None	PMA	HC plasma	iBD plasma	aBD plasma
Neutrophils					
gp91-phox					
HC	23.98 ± 11.55	399.71 ± 47.86^a^	148.26 ± 29.29	261.27 ± 32.52^b^	271.33 ± 44.23^b^
iBD	19.66 ± 11.23	402.00 ± 71.57^a^	156.95 ± 31.23	291.87 ± 45.39^b^	297.35 ± 22.48^b^
aBD	32.68 ± 17.61	464.71 ± 48.45^a^	139.05 ± 32.75	277.88 ± 41.76^b^	278.64 ± 39.95^b^
p22					
HC	20.93 ± 11.40	453.04 ± 70.51^a^	139.25 ± 31.37	262.56 ± 36.43^b^	281.02 ± 53.86^b^
iBD	23.72 ± 12.25	438.87 ± 52.98^a^	132.62 ± 17.28	279.12 ± 38.67^b^	282.62 ± 35.75^b^
aBD	38.82 ± 24.88	422.75 ± 64.76^a^	152.78 ± 9.32	288.74 ± 30.77^b^	314.94 ± 40.60^b^
p40					
HC	22.73 ± 9.96	377.33 ± 115.38^a^	165.77 ± 33.20	279.88 ± 38.13^b^	299.00 ± 49.97^b^
iBD	42.43 ± 7.36	453.37 ± 77.82^a^	133.37 ± 16.08	279.00 ± 50.57^b^	293.12 ± 31.47^b^
aBD	28.85 ± 12.85	475.42 ± 79.97^a^	160.71 ± 20.86	274.71 ± 55.96^b^	253.00 ± 36.56^b^
p47					
HC	22.16 ± 9.23	383.05 ± 69.88^a^	132.90 ± 38.84	247.97 ± 92.38^b^	280.63 ± 59.20^b^
iBD	35.68 ± 22.66	447.75 ± 41.11^a^	151.53 ± 29.26	274.12 ± 50.08^b^	270.12 ± 56.07^b^
aBD	21.04 ± 7.94	400.00 ± 57.01^a^	141.27 ± 34.16	282.12 ± 16.01^b^	293.00 ± 50.48^b^
p67					
HC	32.78 ± 13.00	487.36 ± 93.67^a^	162.11 ± 32.10	288.44 ± 42.48^b^	296.11 ± 55.36^b^
iBD	36.61 ± 15.39	396.50 ± 57.82^a^	145.12 ± 30.69	289.50 ± 46.72^b^	266.12 ± 54.21^b^
aBD	26.42 ± 9.91	425.42 ± 50.71^a^	160.00 ± 22.37	296.71 ± 42.42^b^	274.57 ± 39.45^b^
Monocytes					
gp91-phox					
HC	29.75 ± 16.20	412.02 ± 68.52^a^	154.13 ± 19.00	355.05 ± 107.18^b^	421.50 ± 78.28^b^
iBD	16.43 ± 5.38	415.43 ± 60.54^a^	148.00 ± 33.64	318.06 ± 17.94^b^	330.40 ± 15.98^b^
aBD	22.41 ± 8.09	468.62 ± 30.17^a^	149.41 ± 26.47	263.51 ± 50.51^b^	282.71 ± 50.99^b^
p22					
HC	21.37 ± 12.57	486.14 ± 82.35^a^	157.44 ± 40.22	267.42 ± 34.51^b^	281.82 ± 39.82^b^
iBD	23.00 ± 14.07	442.61 ± 44.55^a^	157.41 ± 30.28	281.65 ± 35.56^b^	283.66 ± 43.68^b^
aBD	27.25 ± 21.68	414.80 ± 33.12^a^	129.20 ± 15.01	272.47 ± 29.72^b^	235.70 ± 53.94^b^
p40					
HC	28.21 ± 10.94	467.00 ± 67.57^a^	147.22 ± 25.00	286.11 ± 37.36^b^	273.77 ± 53.97^b^
iBD	54.53 ± 6.55	560.12 ± 74.73^a^	142.25 ± 30.90	259.25 ± 42.82^b^	289.25 ± 35.70^b^
aBD	45.62 ± 9.24	468.75 ± 78.34^a^	137.00 ± 25.80	290.37 ± 31.87^b^	301.25 ± 44.61^b^
p47					
HC	27.45 ± 11.87	387.52 ± 45.42^a^	155.84 ± 17.92	246.52 ± 37.54^b^	263.57 ± 60.32^b^
iBD	23.56 ± 15.99	416.77 ± 49.75^a^	118.50 ± 27.70	278.71 ± 35.03^b^	286.70 ± 49.01^b^
aBD	29.49 ± 23.42	427.37 ± 63.68^a^	160.71 ± 24.88	264.12 ± 21.63^b^	262.01 ± 38.54^b^
p67					
HC	27.09 ± 17.14	453.77 ± 62.69^a^	160.44 ± 27.99	272.55 ± 42.45^b^	275.22 ± 46.01^b^
iBD	29.01 ± 12.42	420.12 ± 71.30^a^	140.37 ± 26.73	281.75 ± 38.49^b^	303.75 ± 48.17^b^
aBD	29.41 ± 12.61	447.00 ± 67.93^a^	142.50 ± 34.80	249.75 ± 39.07^b^	269.50 ± 33.04^b^

*Abbreviations: aBD* Active Behçet’s disease, *HC* Healthy control, *iBD* Inactive Behçet’s disease, *PMA* Phorbol 12-myristate 13-acetate

Neutrophils and monocytes were obtained from HCs (*n* = 9), patients with iBD (*n* = 8), and patients with aBD (*n* = 8), and protein expression as determined by mean fluorescence intensity was found to be increased after stimulation with BD plasma

Statistical analysis was performed using one-way analysis of variance with the Bonferroni posttest

^a^ *p* < 0.05 compared with unstimulated cells and any other stimulus

^b^ *p* < 0.05 compared with unstimulated cells and stimulation with plasma from HCs

### CD40/CD40L pathway is associated with increased NET release and oxidative burst in patients with BD

**Fig. 5:**
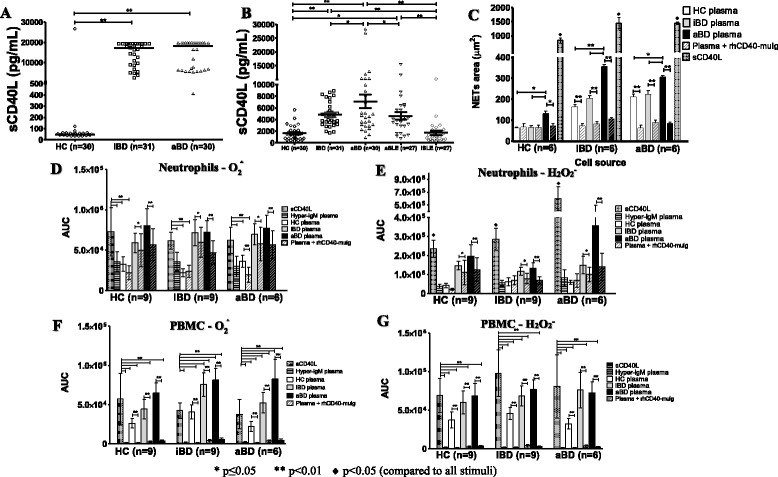
Soluble CD40 ligand (sCD40L) plasma levels are increased in patients with Behçet’s disease (BD) and stimulate neutrophil extracellular trap (NET) release and oxidative burst. **a** Screening with the addressable laser bead immunoassay (*see* Additional file 2: Table S2 for more details) showed strikingly higher sCD40L plasma levels in patients with inactive Behçet’s disease (iBD; *n* = 31) and patients with active Behçet’s disease (aBD; *n* = 30) than in healthy control subjects (HC; *n* = 30). **b** sCD40L plasma levels measured by enzyme-linked immunosorbent assay in HCs, patients with iBD, patients with aBD, and patients with active systemic lupus erythematosus (aSLE; *n* = 27) or patients with inactive systemic lupus erythematosus (iSLE; *n* = 27). **c** Effect of sCD40L or plasma from HCs, patients with iBD, and patients with aBD blocked with recombinant human CD40 (rhCD40-muIg) upon NET release by neutrophils from HCs and patients with BD. **d–g** Superoxide (**d** and **f**) and hydrogen peroxide (**e** and **g**) production by neutrophils (**d** and **e**) and peripheral blood mononuclear cells (PBMCs) (**f** and **g**) after stimulation with sCD40L, CD40L-poor plasma from hyper immunoglobulin M (hyper-IgM) patients, or aBD plasma containing sCD40L-blocking rhCD40-muIg, calculated by the AUC. Bars are defined as follows: **a** Median; **b–g** mean ± SE. Statistical analysis was performed using the Kruskal-Wallis test with the Mann-Whitney posttest (**a**) or one-way analysis of variance with the Bonferroni posttest (**b–g**)

The above results indicate a possible soluble factor carried by the plasma from patients with BD that would be responsible for activating NET release and oxidative burst. In an effort to identify this factor, we determined the concentration of 64 cytokines and 13 soluble receptors in the plasma from patients with BD and from HCs by multiplex technology (Additional file 2: Table S2). Among the several factors that showed the greatest differences between HC and BD plasma, sCD40L was markedly higher in samples from patients with BD (Fig. 5a). This result was confirmed by ELISA, which demonstrated that sCD40L plasma levels from patients with aBD are significantly higher than those from HCs and patients with iBD (Fig. 5b). To gauge the comparative level of CD40L expression in patients with BD, we quantified sCD40L in patients with iSLE and patients with aSLE, whose CD40L expression is known to be highly upregulated in CD4^+^ T cells and even aberrantly expressed on CD8^+^ T cells, B cells, and monocytes [27, 28]. Despite sCD40L levels being markedly elevated in aSLE, the levels observed in aBD were even higher. In patients with iBD, sCD40L plasma levels were comparable to those in patients with aSLE and higher than those in HCs and patients with iSLE (Fig. 5b). It is remarkable that patients with aBD presented a wide range of sCD40L plasma levels, suggesting some heterogeneity in the pathophysiology of the disease. Of note, nonautoimmune diseases previously associated with high levels of sCD40L, such as myocardial infarction [29], atherosclerosis [30], and diabetes mellitus [31], were not observed in our cohort. Furthermore, the distribution of other risk factors for cardiovascular diseases, namely arterial hypertension (iBD = 9.67% vs aBD = 20%; *p* = 0.3) and dyslipidemia (iBD = 22.58% vs aBD = 6.66%; *p* = 0.14), was not different between the two groups of patients.

We then investigated the hypothesis that sCD40L was the stimulating factor responsible for the increased NETosis and oxidative burst after stimulation with BD plasma. In in vitro experiments, we stimulated cells with recombinant sCD40L or with plasma from patients with BD in the presence or absence of rhCD40-muIg used as a specific sCD40L blocking agent. NET release was significantly reduced after sequestering sCD40L with rhCD40-muIg and increased when stimulated with recombinant sCD40L (Fig. 5c). Similarly, superoxide (Fig. 5d and f) and hydrogen peroxide (Fig. 5e and g) production by neutrophils (Fig. 5d and e) and PBMCs (Fig. 5f and g) from patients with iBD, patients with aBD, and HCs was also significantly stimulated by sCD40L and decreased by sCD40L blockade. In addition, sCD40L-poor plasma obtained from patients with CD40L deficiency (hyper-IgM plasma) was not able to increase oxidative burst (Fig. 5d–g).

### CD40L, Mac-1, and phosphorylated NF-κB p65 subunit are overexpressed in patients with BD

**Fig. 6:**
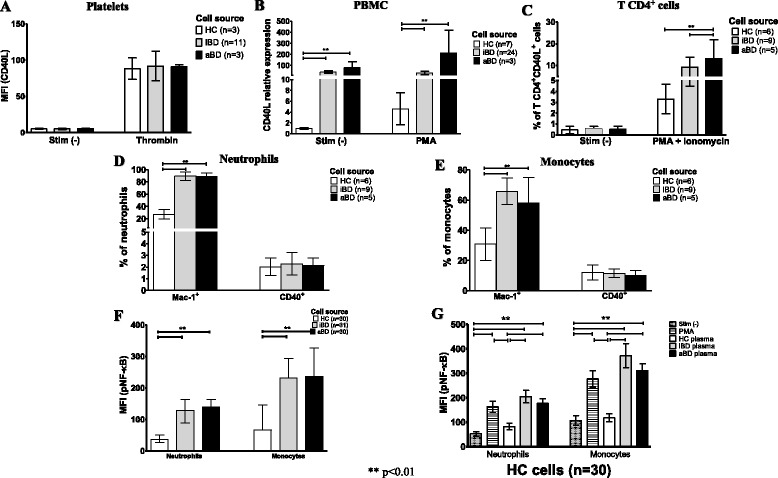
CD40 ligand (CD40L) gene expression is increased in peripheral blood mononuclear cells (PBMCs) from patients with Behçet’s disease (BD). **a** Platelet expression of CD40L (mean fluorescence intensity [MFI]) was determined under basal conditions and after 5 minutes with 0.2 IU/ml thrombin by flow cytometry in healthy control subjects (HCs), patients with inactive Behçet’s disease (iBD), and patients with active Behçet’s disease (aBD). **b** Higher CD40L relative gene expression in PBMCs from patients with BD than in HCs was demonstrated by qRT-PCR, despite an inability to increase CD40L expression in BD cells after 3 h of stimulation with 10 ng/ml phorbol 12-myristate 13-acetate (PMA). **c** A higher number of CD4^+^CD40L^+^ T cells from patients with BD than from HCs after 3-h stimulation with 25 ng/ml PMA + 1.5 μM ionomycin, determined by flow cytometry. **d** and **e** The number of Mac-1^+^ neutrophils was constitutively increased in patients with BD (**d**) and monocytes (**e**), determined by flow cytometry. No difference in CD40 surface expression was observed. **f** Phosphorylated NF-κB p65 subunit expression in resting neutrophils and monocytes from patients with BD was significantly higher than in HCs. **g** Plasma from patients with BD exerted a stronger stimulus on phosphorylated NF-κB p65 subunit expression than HC plasma in normal neutrophils and monocytes. Bars represent mean ± SE. Statistical analysis was performed using one-way analysis of variance with the Bonferroni posttest

To determine the source of the elevated sCD40L in the plasma of patients with BD, we evaluated expression by the normal cell sources of CD40L in peripheral blood. Although platelets are a major source of sCD40L, no difference in CD40L protein expression was observed in platelets from patients with BD compared with those of HCs (Fig. 6a). In contrast, on one hand, CD40L gene expression was strikingly increased in resting PBMCs from patients with BD compared with HCs, but there was not an additional increase after PMA stimulation (Fig. 6b). On the other hand, the number of CD4^+^CD40L^+^ T cells from patients with BD was significantly higher after combined PMA-ionomycin stimulation, suggesting that there is a larger preexisting pool of CD40L protein in T cells (Fig. 6c). Interestingly, contrary to previous observations in patients with lupus [27, 28], CD40L expression on CD8^+^ T cells and monocytes from patients with BD was not different from that observed in HC cells (Additional file 3: Figure S1). Additionally, macrophage integrin Mac-1, but not CD40 protein, was overexpressed in resting neutrophils (Fig. 6d) and monocytes (Fig. 6e) from patients with BD compared with those from HCs. Finally, phosphorylation of the NF-κB p65 subunit was constitutively increased in neutrophils and monocytes from patients with BD, suggesting chronic baseline activation (Fig. 6f) of these cells. Importantly, phosphorylation of the NF-κB p65 subunit was significantly increased in normal neutrophils and monocytes after stimulation with iBD or aBD plasma (Fig. 6g).

## Discussion

In this study, we systematically evaluated several phagocyte functions in BD, thus complementing previous investigations in which researchers assessed individual neutrophil functions in this disease [4, 8, 10, 32]. Previous findings demonstrating the absence of primary activation of BD phagocytes [9] and the stimulating effect of plasma from patients with BD on chemotactic function [10] and oxidative burst [11] of normal neutrophils corroborate our findings that a soluble plasma factor plays a relevant role in the neutrophil hyperactivity observed in BD, which is the main contribution of the present study. NET release by neutrophils, but not the oxidative burst of phagocytes, was increased in nonstimulated cells from patients with BD. Stimulation with BD plasma induced a significant increase in both functions, in some instances comparable to a PMA-induced response. Interestingly, neutrophils from patients with aBD exhibited higher hydrogen peroxide production after stimulation with aBD plasma than other cells or stimuli, suggesting that cells from patients with active disease are in a “preactivated” state.

Several of the evaluated soluble mediators were increased in plasma from patients with BD in comparison to that from HCs; however, the elevation in sCD40L plasma levels in patients with BD was striking. Remarkably, aBD plasma exerted a stronger stimulus on NET release than iBD or HC plasma, correlating with the higher levels of sCD40L in aBD vs iBD. Moreover, the recapitulation of the stimulating effect of BD plasma on phagocyte function by treatment with rh-sCD40L and the inhibition by sCD40L blockade further support our interpretation that sCD40L carried in BD plasma is directly associated with some of the functional abnormalities observed in BD phagocytes. This was further supported by our demonstration that sCD40L-deficient plasma failed to stimulate hydrogen peroxide or superoxide production in similar experiments.

High sCD40L serum levels have been demonstrated in other rheumatic autoimmune diseases [33–36], especially in SLE [37]. By comparing the relative amount of sCD40L present in BD with that in SLE, we found sCD40L plasma levels in aSLE to be comparable to those observed in iBD and significantly lower than those observed in patients with aBD, highlighting the significant background activation of this pathway in BD. Notably, the level of sCD40L expression in patients with iSLE was similar to that observed in HCs. Our data confirm and extend previous findings by demonstrating, in BD cells, the stimulating effect of sCD40L on NET release and oxidative burst and by providing evidence suggesting that this effect is initiated by NF-κB signaling, as previously described in mice [14, 38]. Mac-1 may be secondarily involved in this phenomenon because this adhesion receptor complex, usually upregulated on activated myeloid cells, is overexpressed in resting BD neutrophils [15].

Platelets are the main source of sCD40L, but other cells can express and shed CD40L, including activated T cells. In contrast to the study by Fernández-Bello et al. [14], our study did not show abnormally high CD40L expression by activated platelets from patients with BD; differences in methodology may be the reason for this discrepancy. We used a physiologic stimulus (thrombin activation), whereas Fernández-Bello et al. [14] used a synthetic thrombin receptor-activating peptide assay. In contrast to our observations in platelets, we clearly demonstrate increased CD40L surface expression by in vitro activated CD4^+^ T cells associated with increased basal gene expression by PBMCs from patients with BD. Therefore, our hypothesis is that CD4^+^ T cells from patients with BD constitutively present an enlarged pool of mRNA and preformed CD40L protein, which in turn is quickly expressed at the membrane surface under stress. Ultimately, activated T cells may also contribute to the high sCD40L plasma levels observed in BD. In addition, because BD is primarily a mucocutaneous disorder, fibroblast and epithelial cells, especially those from the uvea, retina, and oral and genital cavities, are also candidates to be sources of sCD40L. Further studies are necessary to test this hypothesis.

The finding that resting phagocytes from patients with BD and HCs did not differ regarding oxidative profile and NADPH oxidase gene and protein expression supports the concept that phagocyte dysfunction in BD is not constitutional. By the same token, our results provide hints about the origin of the factors stimulating phagocytes in BD, because NADPH oxidase expression and oxidative burst activity increased after stimulation with the BD plasma pools. Interestingly, though, sCD40L blocking inhibited superoxide and hydrogen peroxide production less intensely in neutrophils than in PBMCs, suggesting the presence of additional stimulatory serum factors acting on neutrophils. Although the main contribution of the present study relies on showing the original association between sCD40L and phagocyte function in BD, other soluble mediators were slightly but significantly increased in patients with BD, and several others are yet to be described. Moreover, each cell type has distinct responsiveness to different stimuli, which can also explain the differences observed between neutrophils and PBMCs. Additional studies are already being designed by our group in an effort to clarify this question.

Increased NET release has previously been demonstrated in SLE [39], rheumatoid arthritis [40], and antineutrophil cytoplasmic antibody-associated vasculitis [41]. Our study demonstrates increased NET release by resting neutrophils from patients with BD and a further increase after stimulation with aBD plasma, indicating that circulating neutrophils from these patients have undergone some degree of activation. We hypothesize that the proinflammatory environment in BD, especially represented by elevated sCD40L plasma levels, stimulates neutrophil activation and NET release in vivo. Recent studies suggest that NETosis also serves as a physical trail directing cell migration, especially of phagocytes, to inflammatory sites [42]. In view of the current demonstration of increased NETosis in BD, we hypothesize that this mechanism contributes to the increased neutrophil migration [8] and chemotaxis [10] observed in this disease. Further studies should address possible signaling pathways involved in sCD40L-induced NET release and phagocyte migration in BD.

The evidence provided here opens potential opportunities for the development of targeted therapies. A study in a lupus mouse model [43] and a phase I clinical study using a PEGylated anti-CD40L antibody fragment showed promising results [44]. However, previous clinical trials done to determine the efficacy of anti-CD40L therapy in transplantation [45] and immune thrombocytopenic purpura [46] were discontinued because of severe thromboembolic events probably caused by cross-linking of CD40L on platelets.

Interleukin (IL)-8 is a chemoattractant and neutrophil activator and thus would be a candidate plasma factor involved in the phagocyte activation observed in BD. This possibility is favored by pieces of evidence, including increased *IL8* gene expression in macrophages after stimulation with aBD plasma [47] as well as high IL-8 serum [48] and synovial concentrations in patients with BD [49]. Surprisingly, our data did not demonstrate increased IL-8 plasma levels in patients with BD, which may be related to the wide genotypic and phenotypic variation that may occur in distinct ethnic groups, suggesting that various factors associated with sCD40L may be acting in the disease’s pathogenesis. Additionally, the multiplex screening method used may not detect small differences between the groups, constituting an independent bias.

As a pleiotropic and polygenic disease, patient heterogeneity is an important bias. Although clinical and therapeutic confounding variables did not demonstrate any interference with the parameters studied, such factors can never be completely ruled out. It should be noted that colchicine, a medication previously demonstrated to inhibit inflammasome activation [50], NF-κB activation [51], and superoxide production [52], had been equally administered in the iBD and aBD groups. Moreover, our cohort was constituted of a sample of the Brazilian population, which represents a unique blend of European, African, and Native American descendants. Therefore, our findings should be confirmed in studies of other ethnic groups exposed to distinct environmental conditions because our patients may represent only a fraction of the cases and a piece in the BD pathophysiologic enigma.

## Conclusions

The present study shows that plasma from patients with BD exerted a strong stimulus on the production of reactive oxygen species and NET release, and it provides evidence indicating that this effect is induced by sCD40L. In addition, it provides evidence that resting neutrophils from patients with BD display increased NET-releasing activity. These preliminary results suggest new possibilities for further exploring the role of the CD40/CD40L pathway in phagocyte functional abnormalities in BD pathophysiology, and they may suggest novel therapeutic targets for this disease.

## Additional files

Additional file 1: **Table S1.** Genes and primers evaluated by qRT-PCR. (PDF 102 kb)

Additional file 2: **Table S2.** Plasma levels of 64 cytokines and 13 soluble receptors from patients with active (aBD), patients with inactive Behçet’s disease (iBD), and healthy control subjects (HC) determined by addressable laser bead immunoassay. (PDF 249 kb)

Additional file 3: **Figure S1.** CD40L expression is similar on monocytes and CD8^+^ T cells from patients with Behçet’s disease. Percentages of CD8^+^CD40L^+^ T cells (**a**) and CD40L^+^ monocytes (**b**) were determined under basal conditions and after 3-h stimulation with 1.5 μM ionomycin and/or 25 ng/ml PMA by flow cytometry in healthy control subjects (HC), patients with inactive Behçet’s disease (iBD), and patients with active Behçet’s disease (aBD). No difference in CD40L^+^ surface expression was observed. (PDF 200 kb)

## Abbreviations

aBD: Active Behçet’s disease
ANOVA: Analysis of variance
aSLE: Active pediatric systemic lupus erythematosus
BD: Behçet’s disease
cDNA: Complementary DNA
CNS: Central nervous system
ELISA: Enzyme-linked immunosorbent assay
HC: Healthy control
HIgM: Hyper immunoglobulin M
iBD: Inactive Behçet’s disease
IL: Interleukin
iSLE: Inactive pediatric systemic lupus erythematosus
MFI: Mean fluorescence intensity
mRNA: Messenger RNA
NADPH: Nicotinamide adenine dinucleotide phosphate
NET: Neutrophil extracellular trap
PBMC: Peripheral blood mononuclear cell
PE: Phycoerythrin
PMA: Phorbol 12-myristate 13-acetate
PS: Patient with sepsis
rhCD40-muIg: Recombinant CD40
sBR-BDCAF: Simplified Behçet’s Disease Current Activity Form adapted for Portuguese by the Sociedade Brasileira de Reumatologia
sCD40L: Soluble CD40 ligand
SLE: Systemic lupus erythematosus
TNF: Tumor necrosis factor
