# Supplementary material for: Soluble CD40L is associated with increased oxidative burst and neutrophil extracellular trap release in Behçet’s disease
Source: Arthritis Res Ther. 2017 Oct 19;19:235. doi: 10.1186/s13075-017-1443-5 (PMC5649058; doi:10.1186/s13075-017-1443-5)
Supplement: Supplementary file 1 — Table S1. Genes and primers evaluated by qRT-PCR. (PDF 102 kb) [file 13075_2017_1443_MOESM1_ESM.pdf]

Table S1 – Genes and *primers* evaluated by quantitative real-time PCR.

**Protein (*Gene*)**	***Primer* Foward**	***Primer* Reverse**
p22 (*CYBA*)	5'-agg aat tac tat gtt cgg gcc g-3'	5'-cag tag gta gat gcc gct cg-3'
gp91-phox (*CYBB*)	5'-aga ctt tgt atg gac ggc cc-3'	5'-act cag ggt ttc agc caa gg-3'
p47 (*NCF1*)	5'-cct ctt tcc agt gca ttt aag g-3'	5'-gat gtg acg gat gaa ggt gtc-3'
P67 (*NCF2*)	5'-gag gct acc caa cca gag g-3'	5'-aaa tgc cca cct tcc ctt tg-3'
p40 (*NCF4*)	5'-gat gcc tcc tta ctc cag cc-3'	5'-cgt ccc ggt aat tca gag cta t-3'
β-actin	5'- tca ccg agc gcg gct-3'	5'-taa tgt cac gca cga ttt ccc-3'
