# Supplementary material for: Soluble CD40L is associated with increased oxidative burst and neutrophil extracellular trap release in Behçet’s disease
Source: Arthritis Res Ther. 2017 Oct 19;19:235. doi: 10.1186/s13075-017-1443-5 (PMC5649058; doi:10.1186/s13075-017-1443-5)
Supplement: Supplementary file 2 — Table S2. Plasma levels of 64 cytokines and 13 soluble receptors from patients with active (aBD), patients with inactive Behçet’s disease (iBD), and healthy control subjects (HC) determined by addressable laser bead immunoassay. (PDF 249 kb) [file 13075_2017_1443_MOESM2_ESM.pdf]

Table S2 – Plasma levels of 64 cytokines and 13 soluble receptors from active (aBD) and inactive Behçet's disease (iBD) patients and healthy controls (HC) determined by addressable laser bead immunoassay.

Cytokines[a] (pg/mL)	HC (n=30)	iBD (n=31)	aBD (n=30)	p-value
**sCD40L**	47.45/33.68–26,743.00	17,234.00/2,346.00–19,279.00	18,288.50/412.58–19,883.00	<0.01
**IL1α**	17.75/12.24–122.95	23.81/14.38–147.36	20.66/10.59–96.33	NS
**IL1β**	2.54/1.54–6.70	4.13/1.94–20.50♦	0/0–20.33¶	<0.01
**IL-1Ra**	46.87/34.31 – 121.51	63.63/47.01 – 297.79*	62.00/41.30 – 297.19*	<0.01
**sIL1-RI**	29.20/20.72 – 138.72	44.32/0 – 213.99	35.47/0 – 260.86	NS
**sIL1-RII**	5520.64±2532.45¶	9369.78±4212.03*♦	5823.89±1855.61¶	<0.01
**IL2**	3.18/1.94–8.87¶	4.27/2.53–25.85*♦	2.61/0–29.33¶	<0.01
**sIL2-Ra**	991.47±465.06	1459.92±818.41	1297.84±521.24	NS
**IL3**	1.40/0–5.33	1.70/1.38–9.84♦	0/0–7.12¶	<0.01
**IL4**	13.80/9.96–353.44	20.29/11.67–80.18	18.17/8.14–144.49	NS
**sIL4-R**	159.86/57.13 – 354.93¶	501.85/158.97 – 1512.99*♦	203.26/86.16 – 548.21¶	<0.01
**IL5**	3.08/1.80–11.02¶	4.25/2.24–27.18*♦	1.08/0–15.05¶	<0.01
**IL6**	4.10/2.59–8.61¶	8.51/4.29–137.24*♦	5.95/2.38–45.80¶	<0.01
**sIL6-R**	10526.05/4593.32 – 27412.50¶	18122.82/0 – 200000.76*	13179.64/0 – 150969.32	<0.05
**IL7**	4.55±2.53¶	9.79±6.56*♦	4.30±3.71¶	<0.01
**IL8**	10.65/2.91–136.50	14.20/5.01–57.97	18.73/1.31–281.36	NS
**IL9**	2.34/1.23–9.81¶	3.74/2.42–10.64*♦	0.77/0–11.23¶	<0.01
**IL10**	9.78/7.48–30.68¶	15.98/11.63–358.12*♦	10.99/6.24–60.19¶	<0.01
**IL-12p40**	17.01±6.58	35.13±15.01*	37.36±20.75*	<0.01
**IL-12p70**	9.86/6.39–76.36	13.25/8.31–319.10	9.98/6.69–256.30	NS
**IL13**	4.28/2.77–30.67¶	7.40/3.74–43.08*♦	4.50/1.28–20.41¶	<0.01
**IL15**	6.79±1.92¶	10.41±4.13*♦	7.01±3.30¶	<0.01
**IL16**	51.44±38.06	70.19±35.37	68.83±27.41	NS
**IL17**	6.17/2.98–75.56♦	7.49/3.42–200.11♦	3.92/0–542.35*¶	<0.01
**IL20**	26.62/0–181.59♦	30.37/0–86.35♦	51.49/24.16–88.18*¶	<0.01

Cytokines[a] (pg/mL)	HC (n=30)	iBD (n=31)	aBD (n=30)	p-value
**IL21**	7.24/5.30–27.80♦	7.75/0–146.50♦	9.39/7.51–31.76*¶	<0.01
**IL23**	178.00/104.21–629.40	177.45/106.82–4275.00	197.70/123.78–998.14	NS
**IL28A**	47.10/36.89–469.58	51.86/35.10–225.38	55.17/42.30–222.10	NS
**IL33**	11.78/0–52.90	12.57/0–384.81	12.27/0–115.77	NS
**TNFα**	6.88/3.28 – 17.01	14.14/6.00 – 45.36*	11.57/0.00 – 45.28*	<0.01
**TNFβ**	11.34/8.65–847.32	10.94/8.16–71.00	9.12/5.22–34.27	NS
**sTNFRI**	761.68±292.31	1139.27±414.94*	1232.20±414.36*	<0.01
**sTNFRII**	5434.61±1836.53	9997.32±4738.43*	9761.44±6254.79*	<0.01
**TGFα**	3.42/0–11.09	3.84/0–38.37	5.11/0–17.73	NS
**IFNα**	20.15/14.83 – 69.73	39.93/16.41 – 166.58*	42.67/24.66 – 132.68*	<0.01
**IFNγ**	8.79/5.81 – 40.73	14.31/6.90 – 155.96*	17.97/5.31 – 198.54*	<0.01
**IP10**	168.49/48.16 – 621.48	532.99/199.27 – 1642.00*	372.38/128.66 – 3729.00*	<0.01
**MIP1α**	10.13/4.69 – 39.15	11.09/4.33 – 119.89♦	22.12/10.92 – 193.19*¶	<0.01
**MIP1β**	16.26/6.34 – 42.36	23.74/8.58 – 146.82*	26.38/12.00 – 88.63*	<0.01
**MIP1∆**	3649.00/774.24–18757	3796.00/543.59–10174	3429.00/196.72–19369	NS
**Eotaxin-1**	21.05/14.31 – 626.10	417.27/22.15 – 1546.00*	341.92/39.13 – 1340.00*	<0.01
**Eotaxin-2**	763.87/155.49 – 5681.00	242.06/46.82 – 1463.00*	236.07/81.46 – 2108.00*	<0.01
**Eotaxin-3**	52.47/0–159.78	58.83/0–179.88	57.05/26.78–196.30	NS
**BCA-1**	11.97/5.65 – 54.49	25.34/14.03 – 2210.00*	38.00/18.63 – 309.40*	<0.01
**CTACK**	243.16/51.19 – 1123.00	608.99/208.13 – 976.66*	694.43/287.05 – 1304.00*	<0.01
**RANTES**	14206.96±4453.02	8845.67±2569.96*	9127.13±3743.69*	<0.01
**TRAIL**	53.40±22.86	64.47±22.19	55.44±21.97	NS
**FGF**	64.98/51.31 – 142.43	81.78/60.28 – 1147.00*	86.43/60.83 – 317.37*	<0.01
**G-CSF**	58.23/40.37 – 165.64	74.64/47.63 – 314.22*	67.76/46.24 – 967.36*	<0.01
**EGF**	86.23/20.27 – 878.41	41.94/18.01 – 243.74*	40.29/17.84 – 227.78*	<0.01
**GM-CSF**	17.59/11.92–44.56	19.83/14.61–142.31	15.53/8.34–104.87	NS
**PDGF-AA**	1836.50/282.16–5825	1539.00/539.50–4268	1273.50/134.45–4527	NS

Cytokines[a] (pg/mL)	HC (n=30)	iBD (n=31)	aBD (n=30)	p-value
**PDGF-AB/BB**	8290.40/2194–17759	8974.00/1340–20905	9372.00/1150–21857	NS
**SCF**	34.85/23.74–75.72	42.66/28.19–153.67	40.56/22.04–77.87	NS
**VEGF**	205.17/134.60–440.13	207.61/148.52–1923.00	213.72/131.10–3376.00	NS
**sVEGFR1**	336.22/96.03 – 862.13	1724.85/0.00 – 4154.77*	1857.94/99.30 – 3740.25*	<0.01
**sVEGFR2**	19676.71/13388.11 – 27752.86¶	26774.39/15764.65 – 104368.35*♦	21205.25/8240.85–98000.73¶	<0.01
**sVEGFR3**	194.62/29.73 – 1173.59	588.47/0.00 – 3385.05*	609.44/20.03 – 2671.95*	<0.01
**Thrombopoietin**	418.24/227.48–882.36	347.28/242.92–8237.00	406.43/222.13–2778.00	NS
**TSLP**	2.98/0–24.83	0/0–68.05	4.29/0–36.43	NS
**LIF**	9.16/4.68–26.63	7.54/0–46.49♦	12.59/7.58–27.43¶	<0.01
**SDF-1α+β**	1634.12±592.77¶	3225.83±923.98*♦	1383.92±787.63¶	<0.01
**α-GRO**	653.29/221.74–3966.00	594.85/215.69–1417.00	733.83/203.56–3747.00	NS
**MCP-1**	360.26±283.32	384.42±158.58	351.65±258.39	NS
**MCP-2**	24.63/13.10–68.63	24.98/16.98–45.63	25.03/14.99–88.91	NS
**MCP-3**	21.41/15.60–78.73	26.07/17.54–128.04	27.57/18.42–67.26	NS
**MCP4**	47.02/28.91–110.76¶	116.87/35.10–279.25*♦	47.94/25.09–314.91¶	<0.01
**Fractalkine**	59.15/41.68–198.57♦	58.94/45.90–302.91♦	127.07/56.35–402.24*¶	<0.01
**Flt-3L**	55.89/29.63–87.37	54.73/31.08–459.33	43.68/31.08–141.78	NS
**ENA-78**	468.77/88.65–4921.00	471.47/105.78–2515.00	446.81/54.94–8428.00	NS
**TARC**	26.89/3.25–278.16	24.93/4.49–111.62	16.16/4.25–388.75	NS
**6Ckine**	363.59/225.31–1065.00¶	546.74/247.26–976.26*♦	342.75/262.83–729.32¶	<0.01
**CCL1**	4.91/4.10–12.72	5.48/3.59–34.86	5.53/4.29–11.65	NS
**CCL22**	643.34±273.73	561.68±204.79	511.75±345.07	NS
**sCD30**	17.49/0 – 99.00¶	371.89/30.16 – 993.99*♦	22.87/7.61 – 218.44¶	<0.01
**sgp130**	78160/42801 – 100000¶	274510/46460–1210000*♦	78518.83/0 – 100000¶	<0.01
**sRAGE**	237.62/32.12 – 788.06	1388.95/145.39 – 6253.66*	1474.33/187.76 – 3950.32*	<0.01

Legend: 6Ckine: chemokine with 6 cysteines; α-GRO: α-oncogene related growth; BCA-1: B-cell chemokine attracting 1; CTACK: cutaneous T-cell attracting chemokine; EGF:

epidermal growth factor; ENA-78: epithelial neutrophil activating peptide 78; FGF: fibroblast growth factor; Flt-3L: FMS-like tyrosine kinase 3 ligand; G-CSF: granulocytes colonies stimulating factor; IL1Ra: IL1 receptor antagonist; IP10: IFNγ-induced protein 10; LIF: leukemia inhibitor factor; MCP: monocyte chemotactic protein; MIP1: macrophage inflammatory protein 1; PDGF: platelet-derived growth factor; RANTES: regulated on activation, normal T cell expressed and secreted; SCF: stem cell factor; SDF-1α+β: stromal cell-derived factor 1α and 1β; sIL1-R: soluble IL1 receptor; sIL2-Rα: soluble IL2 receptor α; sIL4-R: soluble IL4 receptor; sIL6-R: soluble IL6 receptor; sgp130: soluble glycoprotein 130; sRAGE: soluble receptor of advanced glycosylation end product; sTNFR: soluble TNF receptor; sVEGFR: soluble vascular endothelial growth factor receptor; TARC: thymus and activation-related chemokine; TNF: tumor necrosis factor; IFN: interferon; TRAIL: TNF-related apoptosis-inducing ligand; TSLP: thymic stromal lymphopoietin.

[a] Data are expressed as median/minimum-maximum or mean±standard-deviation, according to normality.

Statistical analysis: Kruskal-Wallis test (post-test: Mann-Whitney) or one-way ANOVA (post-test: Buonferroni) when appropriated.

* $p<0.05$ compared to HC.

¶ $p<0.05$ compared to iBD.

♦ $p<0.05$ compared to aBD.
