# Supplementary material for: Soluble CD40L is associated with increased oxidative burst and neutrophil extracellular trap release in Behçet’s disease
Source: Arthritis Res Ther. 2017 Oct 19;19:235. doi: 10.1186/s13075-017-1443-5 (PMC5649058; doi:10.1186/s13075-017-1443-5)
Supplement: Supplementary file 3 — Figure S1. CD40L expression is similar on monocytes and CD8+ T cells from patients with Behçet’s disease. Percentages of CD8+CD40L+ T cells (a) and CD40L+ monocytes (b) were determined under basal conditions and after 3-h stimulation with 1.5 μM ionomycin and/or 25 ng/ml PMA by flow cytometry in healthy control subjects (HC), patients with inactive Behçet’s disease (iBD), and patients with active Behçet’s disease (aBD). No difference in CD40L+ surface expression was observed. (PDF 200 kb) [file 13075_2017_1443_MOESM3_ESM.pdf]

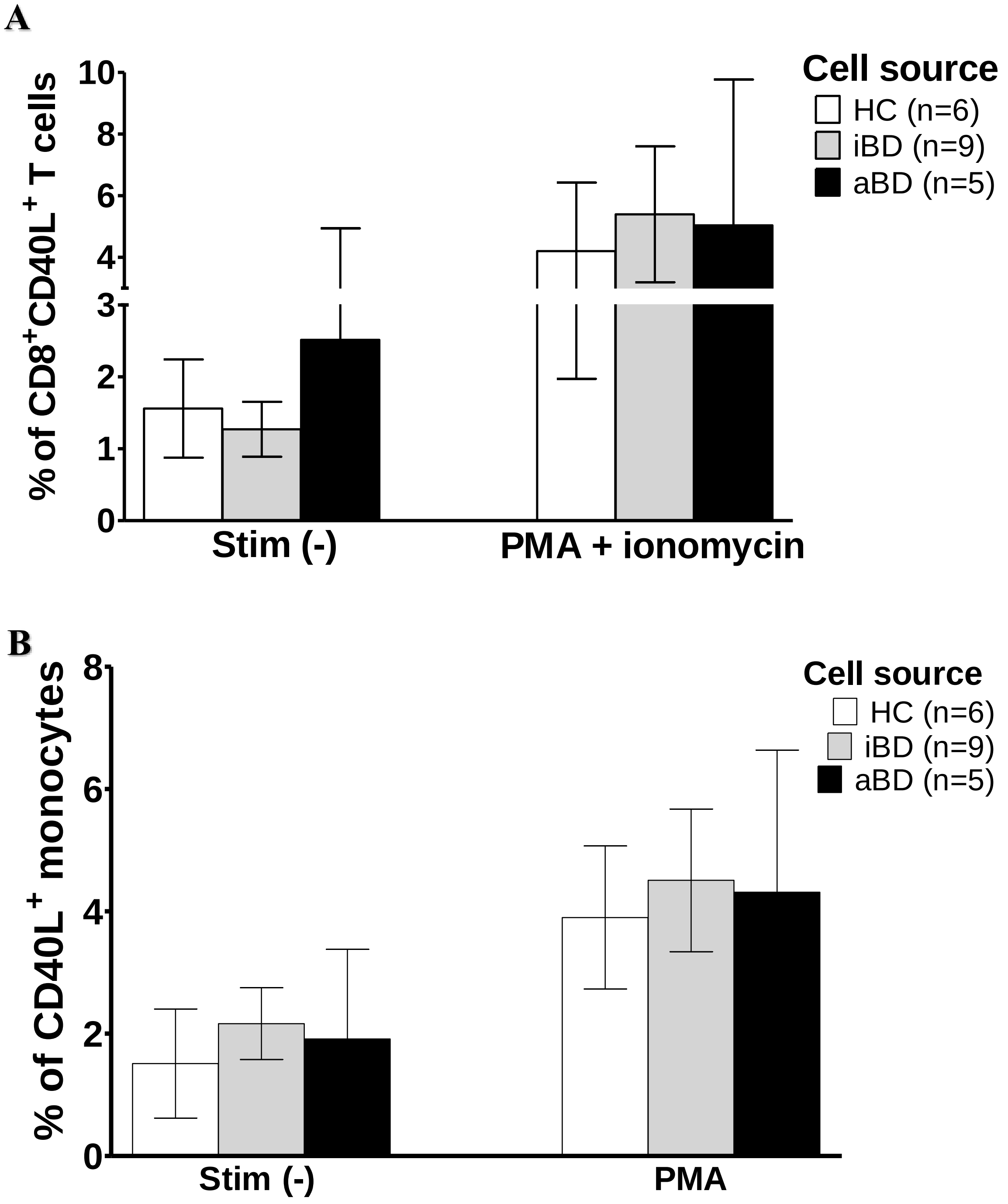

Figure S1 – CD40L expression is similar on monocytes and CD8$^+$ T cells from Behçet's disease patients. Percentages of CD8$^+$CD40L$^+$ T cells (A) and CD40L$^+$ monocytes (B) were determined under basal conditions and after 3h stimulus with 1.5µM ionomycin and/or 25ng/mL PMA by flow cytometry in healthy controls (HC), inactive (iBD) and active Behçet's disease (aBD) patients. No difference in CD40L$^+$ surface expression was observed.
